# A mixed methods protocol to develop a mental health awareness program among adolescents in rural based schools of Mopani District, South Africa

**DOI:** 10.3389/fpubh.2025.1639345

**Published:** 2025-10-02

**Authors:** Tshisikhawe Mahada, T. G. Tshitangano, M. H. Mphasha

**Affiliations:** Department of Public Health, Faculty of Health Sciences, University of Limpopo, Polokwane, South Africa

**Keywords:** adolescents, awareness, disorders, mental health, programs, rural secondary schools

## Abstract

**Background:**

Adolescence is a critical transitional phase characterized by rapid developmental changes, which makes this age group more vulnerable to mental health challenges and disorders. Despite the South African governments’ effort to implement the Integrated School Health Policy (ISHP), the prevalence of depression, anxiety, and suicidal behavior continues to rise among adolescents in rural based secondary schools. Therefore, this protocol aims to develop an intervention program to promote mental health awareness among adolescents in rural based secondary schools of Mopani district, Limpopo province, South Africa.

**Methods:**

The study will be conducted in three phases. Phase 1 will involve a quantitative approach (phase 1A), using a cross-sectional descriptive research design. Cluster sampling method will be used to select schools, followed by systematic sampling to select adolescents between 12 and 18. Simple random sampling will be used to select teachers. Structured questionnaires will be used to collect data that will be analyzed using Statistical Package for Social Sciences software version 24 for data analysis. Qualitative approach (phase 1B) using explanatory research design will follow, which will be dependent on quantitative data. Both purposive and Snowball sampling methods will be utilized to sample adolescents and teachers. Semi-structured interviews will be used to collect data that will be analyzed thematically. Phase 1c will involve the synthesis and integration of the study through strength, weakness, opportunities, threats analysis; where findings from both phases will be consolidated and synthesized into a cohesive whole. Phase 2 will involve the development of a program using a logic model. The program will be presented in a table format, outlining specific objectives, activities, and outcomes. Phase 3 will focus on the validation of the developed program using the Delphi technique. The study has been approved by the University of Limpopo Research Ethics Committee: TREC/189/2025: PG.

**Discussion:**

This research addresses the Sustainable Development Goals (SDGs) 3 and 4, ensuring healthy lives and promoting inclusive education. The study’s findings will contribute to the development of a tailored program to promote mental health awareness among adolescents in rural-based secondary schools. The study’s results will also provide valuable insights for policymakers, educators, and mental health professionals.

**Conclusion:**

The conclusion and recommendations will be based on the findings of the study.

## 1 Introduction

Globally, mental health disorders are a major health concern, with adolescents experiencing high risks of disability, morbidity, and mortality (1). Around 15% of young people aged 10–19 experience a mental health disorder, representing 13% of the global disease burden for this age group (2). In sub-Saharan Africa, 14.3% of children and adolescents face significant psychological challenges, and 9.5% meet criteria for psychiatric diagnoses. In South Africa, mental disorders impact 15–17% of adolescents, with about half of these disorders beginning before age 14 (3). A recent study on youth suicidal behavior in South Africa revealed that approximately 22% of black South African adolescents experience suicidal ideation or attempts (4).

According to (5), risk factors such as low self-esteem, low self-efficacy, and high trait perfectionism can negatively affect adolescent mental development, often leading to partial self-concepts and unrealistic expectations. Bitsko et al. (6) discovered that adverse family conditions, including negative childhood experiences, poor economic status, and parental psychological control, along with peer issues like bullying, social media influence, and lack of support, increase the risk of depression, anxiety, and behavioral problems. Schools are obliged to establish school-based support teams, which may include learner support agents to assist adolescents facing barriers to learning, in collaboration with other schools and community partners (7). However, for many adolescents, these policies and provisions are insufficient to protect their mental health. Poor policy implementation, limited resources, and various overlapping challenges such as substance use, adolescent pregnancy, and domestic violence further complicate matters.

To address adolescent mental health issues, the South African government launched the Integrated School Health Policy (ISHP) in 2012 (4). The school-Based Support Team (SBST) which comprises life orientation (LO) teachers, health team members, school governing body (SGB) members, and peer educators, manages the implementation under the principal’s direction. Despite the introduction of the ISHP to improve mental health awareness, its implementation in rural schools remains inadequate particularly in the Mopani District, Limpopo Province. Many schools in this region lack regular visits from health practitioners, resulting in delayed screening and intervention for adolescents experiencing mental health challenges (8). Furthermore, the HASHTAG program, launched in 2021, aimed to promote and empower adolescent mental health through awareness and support initiatives. However, its impact has been limited by challenges such as inadequate resources, insufficient support teams, and broader social issues like substance abuse and domestic violence (7). While some studies have explored mental health awareness among adolescents and educators, formal and sustainable empowerment programs, especially in rural areas remain limited (9).

Findings continue to reveal that depression affects 19.7% of adolescents, which, while lower than anxiety, is still concerning as it surpasses the 10% prevalence rate among adults (4). Therefore, development of this program is needed to provide guidance in the form of activities and screening tools and direction for promoting mental health awareness and emphasize the need to incorporate mental health education into the curriculum and enhance support services in Mopani district.

## 2 Conceptual framework

### 2.1 PRECEDE–PROCEED model

The PRECEDE–PROCEED model will be used as a conceptual framework that will guide the study. This model offers a framework to systematically arrange various factors and guide the development of targeted interventions aimed at social, behavioral, and environmental change (10). Moreover, this model helps to prioritize a problem, analyze and respond to the associated need and provides a catalyst for change. According to Green and Kreuter (11), the PRECEDE model was developed in the 1970s meaning Predisposing, Reinforcing, and Enabling Constructs in Educational/Environmental Diagnosis and Evaluation. Later, PROCEED was added in recognition of the significance of environmental factors in shaping health and health behaviors. PROCEED stands for Policy, Regulatory, and Organizational Constructs in Educational and Environmental Development (12). This model comprises 8 phases, however, the researcher will focus on only five phases that will be presented as follows.

### 2.2 Needs assessments

**Phase A**: Social Assessment: This phase primarily focuses on identifying desired results and evaluating readiness for change while considering subjective concerns about the health issue. Therefore, in this study mental health problem analysis and identification of health program’s desired results.

**Phase B:** Epidemiological Assessment: In order to identify and prioritize change targets, this phase focuses on identifying (proximal, interpersonal, and distal) behavior as well as (direct/indirect) environmental concerns. The researcher will identify the mental health determinants of the identified problem and set priorities and goals.

**Phase C:** Behavioral and Environmental Assessment: This phase focuses on identifying the behavioral and environmental factors that contribute to the health problems identified in Phase 2. This phase in this study will involve the Identification of behavioral, environmental, and biological factors that impact mental health.

**Phase D:** Educational and Ecological Assessment: This phase’s primary goals are to identify and prioritize change targets while also identifying the predisposing, reinforcing, and enabling factors impacting behaviors and environmental situations established in previous phases. The researcher will Identify predisposing, enabling, and reinforcing factors, which, if modified, could result in behavioral mental health change.

**Figure 1:**
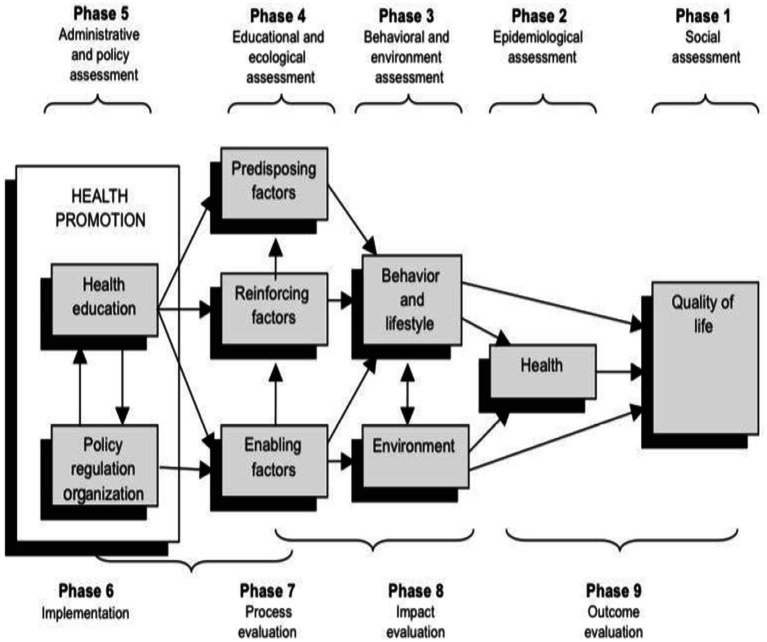
PRECEDE–PROCEED planning model adopted from Classen et al. (32).

**Phase E:** Health program and policy development: this phase focuses on identifying administrative and policy factors that may influence what can be developed. This involves developing and implementing health programs and policies based on the assessments conducted in the previous phases. In this study the researcher will develop a program to promote mental health awareness among adolescents in Mopani district, Limpopo province using logic model (see details in phase 2; Figure 1).

## 3 Methods and study design/s

This study will employ a mixed-methods research approach. Timans et al. (13) define mixed methods as a methodology that integrates both qualitative and quantitative research techniques within a single study to effectively address the research questions. The researcher chose mixed methods in case gaps arise after analyzing quantitative data. Phase 1B, which will follow quantitative approach will help in explaining phase 1A’s findings. Thus, an explanatory sequential mixed methods design will be employed in this study, where the research process will begin with collecting quantitative data, followed by qualitative data (14). The researcher chose explanatory sequential mixed-method design because it offers a two-phase approach where if gaps arise after analyzing quantitative data, the qualitative data will follow to cover gaps and explain findings in detail. Phase 1A of the study will be quantitative approach, using cross-sectional descriptive research design. Based on the need for explanations of findings from quantitative approach, research questions and objectives will be developed to inform phase 1B which will be qualitative approach. Explanatory research design will be used in phase 1B. The researcher will use this research design to understand the relationship between variables or a certain phenomenon that could arise after analyzing quantitative data. The synthesis of findings (will be done using SWOT analysis). Phase 2 will be the development of a program to promote mental health awareness among adolescents in rural based secondary school of Mopani district, Limpopo province using LOGIC model. Phase 3 will focus on the validation of the developed program using the Delphi Technique.

### 3.1 Study setting, population, inclusion and exclusion criteria

#### 3.1.1 Study setting

The study will take place in the Mopani District of Limpopo Province, South Africa, a Category C municipality located in the northeastern part of the province, near Zimbabwe. Covering 20,011 km^2^ and with a population of around 1,092,507, the district features diverse educational environments and infrastructure. It includes 239 public secondary schools across five municipalities: Greater Giyani, Greater Letaba, Greater Tzaneen, and Ba-Phalaborwa. The educational system is divided into two circuits: Mopani East and Mopani West. The researcher selected Mopani District due to its rural nature and limited mental health infrastructure, highlighting the need for tailored interventions for such communities.

#### 3.1.2 Study population

The population of the study will include adolescents between 12 and 18 in Mopani district schools. Adolescents between 12 and 18 were chosen to include all secondary school grades, as this ensures the inclusion of adolescents from early to late adolescence stage. Teachers will also form part of the study.

### 3.2 Inclusion and exclusion criteria

#### 3.2.1 Inclusion and exclusion criteria (leaners)

**Inclusion criteria**: Adolescents of all genders will be included. Adolescents enrolled in a rural-based secondary school within the Mopani District, Limpopo Province in the year of data collection will also form part of the study. Adolescents who have provided informed consent, along with parental or guardian consent if the learner is under 18 years old will also be included.

**Exclusion criteria:** Adolescents with severe mental health conditions require specialized clinical intervention because their participation would affect their mental health state. Adolescents with an attendance rate below 75% in the current academic year, which might hinder consistent participation.

#### 3.2.2 Inclusion and exclusion criteria (teachers)

**Inclusion Criteria**: Teachers with at least 2–3 years of experience working with adolescents in rural schools.

**Exclusion criteria:** Temporary teachers and those unavailable to attend.

**Recruitment:** After obtaining ethical clearance and permission/s to collect data from Limpopo and the Department of Education, the researcher will schedule appointments with the district offices and school principals to request formal permission to collect data from their schools. Appointments will be made with teachers and adolescents so that they will be aware of the study that will be conducted in their schools. Informed consent, assent forms as well as information sheet will be explained for better understating the targeted participants. The researcher will provide participants with pens to complete the questionnaire. The researcher will ensure that the questionnaires are fully completed before submission. The hard copies of the questionnaire will be kept in the locker accessible by the researcher at the researcher’s place of residence. The soft copy data of the respondents that will be shared with the supervisors for verification will be stored in the storage device (USB) that will only be accessed by the researcher and supervisors. Furthermore, the file in question will be protected by a password known by the researcher only. Before completing the questionnaire, participants will be encouraged to complete all applicable questions in full and to complete the questionnaires independently, without assisting each other. The completion of questionnaires will take place at school where seating arrangements will make it possible for participants to sit far apart from each other to avoid copying each other.

**Phase (1a): Quantitative approach.**

This phase will be guided by the following objectives:

**Learners’ objectives:**

- To assess the prevalence and distribution of four most common mental health disorders (depression and anxiety, substance use disorder and post-traumatic stress disorder (PTSD)) among adolescents in Mopani District, Limpopo Province.
- To identify risk factors associated with poor mental health outcomes among adolescents in Mopani district, Limpopo province.

**Teachers’ objectives:**

- To evaluate the level of awareness among teachers regarding mental health issues commonly affecting adolescents in rural-based secondary schools.
- To assess teachers’ perceptions of the support and resources required to address learners’ mental health challenges.

### 3.3 Sampling/selection

#### 3.3.1 Sampling of schools

Cluster sampling will be used to select schools within the Mopani District, which includes about 239 secondary schools. The process will involve developing clusters based on geographical sub-districts in Mopani, which include Ba-Phalaborwa, Greater Giyani, Greater Letaba, Greater Tzaneen, and Maruleng. These sub-districts will be treated as clusters to ensure comprehensive coverage and to avoid bias. Schools within each sub-district will be listed and sampled, with the number of schools to be sampled determined using the formula below, based on the total number of schools in each sub-district (Table 1).


$$\begin{array}{l} \text{Number of schools to}\;\mathrm{be}\;\text{sampled}\;\mathrm{per}\;\mathrm{sub}-\text{district}\\ =\frac{\text{Total}\;\;\text{nmber of schools}\;\mathrm{per}\;\mathrm{sub}-\text{district}}{\text{Number of clusters}} \end{array}$$

**Table 1:** Sampling frame.

Sub-districts within Mopani district	Total number of schools per subdistrict	Sampled schools
Ba-Phalaborwa	15/5	3
Greater Giyani	58/5	12
Maruleng	24/5	5
Greater Tzaneen	62/5	12
Greater Letaba	84/5	17
Total	243/5	49

After conducting cluster sampling, the researcher used a random sampling method to select schools from each cluster. An Excel random number generator was utilized for this purpose. The total number of schools was organized in a spreadsheet for each cluster, and then the number generator was applied.

#### 3.3.2 Sampling/selection of adolescents

Adolescents will be selected systematically. The following steps as highlighted by Latham (15) will be followed when employing a Systematic sampling method: In this study, the population of interest will include all adolescents between 12 and 18 from the sampled schools. The sample size will be determined using Slovin’s formula, after which the researcher will obtain learners’ enrollment records to list the defined population. Each learner on the list will be assigned a consecutive number from 1 to the last entry. The sampling interval will then be calculated by dividing the total population size (N) by the desired sample size (n). Using a random number table, the first unit will be selected, and subsequent units will be chosen at the predetermined interval until the required size is reached.

The sample size for this study will be determined using the sample size formula and taking into consideration available resources and required time to complete the study. The sampling size will be calculated using Slovin’s (16) formula cited in Guilford & Frucher (17). The sample size is given by *n* = N.


$$\left[1+N\;{\left(e\right)}^{2}\right]$$

Where *n* = sample size

*N* = Total number of populations.

e = margin of error, which is 0.05.

In this study *n* = N.


$$\left[1+N\;{\left(e\right)}^{2}\right]$$

Therefore, the following formula will be used to calculate the interval 9(nth)


$$\text{Sampling interval}\;(K)=\frac{\text{Total population}}{\text{Sample size}}$$

#### 3.3.3 Sampling/selection of teachers

Simple random sampling will be used to sample teachers. After obtaining approval for data collection, the researcher will start by collaborating with school principals or the district education office to compile a comprehensive list of permanent teachers in the selected schools. Once the list is finalized, the researcher will assign each teacher a unique identification number to facilitate the random selection process. An Excel random number generator will be utilized to achieve this sampling method.

### 3.4 Data collection

#### 3.4.1 Measurement instrument

Structured questionnaires will be used for data collection. The researcher will develop the questionnaires, which will consist of sections assessing adolescents’ knowledge, attitudes, behaviors, and awareness regarding mental health, as well as symptoms of common mental health problems. The questionnaires will be constructed in English and translated into the main local languages spoken in Mopani District (Xitsonga and Northern Sotho).

### 3.5 Data analysis

Data analysis involves organizing and structuring the information collected from participants and interpreting the extensive data to extract meaningful insights (18). Once data collection is complete, the data will be recorded, followed by a cleaning process to eliminate any invalid questionnaires before analysis. Empty, incomplete, or damaged questionnaires will be identified and documented accordingly.

The researcher will use SPSS (Statistical Package for Social Sciences) software version 24 for data analysis. The researcher will keep record of incomplete and damaged questionnaires so they can be excluded to avoid skewing results. The researcher will start by cleaning the data, followed by coding variables, entering the data into Excel transferring it to SPSS software, and generating frequencies. Descriptive statistics will be used to summarize data. The Chi-square test will be employed to assess the relationship between categorical variables, such as comparing gender differences in adolescents’ awareness of mental health issues. Chi-square refers to a statistical technique used to assess whether there is a meaningful relationship between categorical variables (19). If both variables are continuous, Pearson’s correlation coefficient will be used to assess the strength and direction of the linear relationship between learners’ exposure to mental health education and their awareness scores. If the exposure variable is categorical, associations will instead be assessed using Chi-square tests. According to Shuttleworth (20), the correlation coefficient provides a quantifiable measure of the relationship between two continuous variables, appearing as a single numerical value that can be interpreted to understand the nature and strength of their association. The data analyzed will be presented in charts and graphs.

### 3.6 Ensuring study rigor

#### 3.6.1 Reliability

Reliability refers to the degree to which a research instrument produces consistent results each time it is administered (21). To ensure reliability, the researcher will use test–retest reliability (piloting). In test–retest reliability, the same research instrument will be administered to the same group of respondents between two points in time to determine the consistency of the responses. Test–retest reliability, also known as the coefficient of stability, measures the consistency of scores over time, with a value of 1 indicating perfect reliability and values above 0.9 considered excellent (20). Coefficients between 0.8 and 0.9 are deemed good, 0.7 to 0.8 acceptable, 0.6 to 0.7 questionable, 0.5 to 0.6 poor, and below 0.5 unacceptable (20). To determine the reliability of the research instrument, the researcher will administer the questionnaire twice first in a different school with similar participants a week before actual data collection, and then again after 1 week. The Pearson Correlation Coefficient will be used to calculate reliability.

#### 3.6.2 Validity

Validity refers to the extent to which a research instrument measures what it is intended to measure (22). To ensure face validity, the researcher will present the questionnaire to supervisors for review, while content validity will be ensured by aligning the questions with the research objectives and consulting relevant literature and existing instruments. Experts such as psychologists and registered counselors will also review the questionnaire for relevance and comprehensiveness. Based on their feedback, the questionnaire will be refined and pilot tested to ensure clarity and effectiveness.

### 3.7 Pre-testing

Before beginning the real process of collecting data, the researcher will pre-test the questionnaire. Questionnaires will be administered to adolescents who meet the inclusion criteria. Participants will be sampled through snowball sampling where a teacher will be asked to select/pick a leaner to participate. To make sure the questionnaire accurately measures the research variables, it will be corrected and modified based on the findings of the pre-testing of the instruments. For pre-testing, 5% of the entire sample drawn from various strata will be used. Those who are chosen for pre-testing will not be included in the primary research.

### 3.8 Phase (1b): qualitative approach

This phase will be dependent on the need for explanation of the findings from phase 1(a). Such explanation will require exploration from participants for answers. Therefore, the research question and objectives will only be available after analysis of data from Phase 1(b).

#### 3.8.1 Sampling

##### 3.8.1.1 Sampling/selection of adolescents

Both purposive and Snowball sampling methods will be utilized. An initial participant who meets the selection criteria will be asked to recommend other adolescents who might provide valuable insights. This would help to reach participants who may provide valuable insights into the phenomenon and relationship between variables to be explained.

##### 3.8.1.2 Sampling/selection of teachers

Purposive Sampling will be utilized to sample teachers with the most relevant knowledge or experience regarding mental health, preferably life orientation teachers. Then Snowball Sampling will be employed once the researcher has initial participants, they will be asked to recommend other teachers who might have valuable insights or experiences related to mental health awareness.

#### 3.8.2 Sample size

The study will include all five sub-districts within the Mopani district of Limpopo province. From these, schools will be selected randomly (an Excel random number generator will be utilized). Five adolescents will be sampled from each school, and one teacher will be sampled per school as well, resulting in a total sample size of 30. The final number of participants may be adjusted based on data saturation.

### 3.9 Data collection

#### 3.9.1 Data collection tool

The researcher will use semi-structured interviews to gather information from participants. Interviews will be guided by the objectives and questions that would be formulated after quantitative data collection and analysis if there are gaps that needs in-depth understanding.

#### 3.9.2 Pre-test

Before beginning the real process of collecting data, the researcher will pre-test the interview guide. This will be done to evaluate and refine questions for clarity and relevance, it will be corrected and modified based on the findings of the pre-testing of the instrument. For pre-testing, 1 interview will be conducted per school and participants will be sampled through snowball sampling where a teacher will be asked to select/pick a leaner to participate. Those who are chosen for pre-testing will not be included in the primary research.

### 3.10 Plan for data management and analysis

Thematic data analysis will be used to analyze the information that is gathered from the participants during the interviews. Thematic analysis is a process for methodically finding, classifying, and providing context for meaningful patterns (themes) inside a dataset (23).

Step 1: Familiarization with Data.

The researcher will read through the data and listen to audio recording multiple times to gain an overall understanding of the content.

Step 2: Initial Coding.

The researcher will initiate the process of coding the data. This will be accompanied by labeling or tagging segments of the data (e.g., sentences, paragraphs) with descriptive codes that capture the essence of the content.

Step 3: Generating Themes.

After coding the researcher will look for patterns and connections among the codes. Group related codes together to form preliminary themes.

Step 4: Reviewing Themes.

The identified themes will be reviewed and refined, ensuring that each theme is distinct, meaningful, and supported by coded data.

Step 5: Defining and Naming Themes.

The researcher will define each theme clearly, describing what it represents based on the data. Give each theme a descriptive and meaningful name that summarizes its content.

Step 6: Analyzing and Reporting.

After defining and naming themes, the researcher will analyze themes in relation to her research questions and objectives. Then finally report findings by presenting the identified themes, supporting quotes and interpretations that explain the significance of each theme in the context of your study.

### 3.11 Ensuring trustworthiness

Trustworthiness will be ensured using the following four elements: (1) credibility, (2) conformability, (3) transferability, and (4) dependability. Each element will be explained in detail and the research will explain how the elements will be applied.

- Credibility

Credibility refers to the trust placed in the authenticity or precision of research findings. It ensures alignment between the participants’ viewpoints and the researchers’ interpretation and representation of those views (24). To establish credibility in this study, the researcher will employ analytical triangulation which is also known as peer debriefing and member checks.

- Analytical triangulation/peer debriefing

After completing the research, the findings will be presented to peers for review to enhance objectivity and improve research quality. This peer review process involves constructive feedback on the study’s background, data collection, organization, analysis, and results, and will include input from fellow researchers, colleagues, academic staff, and members of the dissertation committee.

- Member checks

The researcher will return the data to participants to ensure if it accurately reflects their experiences, allowing them to suggest or request changes if the interpretations are unsatisfactory.

- Transferability

Morrow (25) describes transferability as the extent to which readers can apply the findings of a study to their own contexts and consider how broadly the researcher’s theories or assumptions can be generalized. The researcher will assess whether the findings are applicable beyond the specific context of the study. To ensure transferability, the researcher will employ thick description. The researcher will provide thick description by thoroughly outlining all research procedures, from data collection to the final report. Additionally, the researcher will offer comprehensive details about themselves as an instrument, the participants, and the researcher-participant interactions to help readers determine how the findings might be applicable in other contexts.

- Dependability

Dependability refers to the consistency and thorough documentation of the research process, as described by De Vos et al. (26). In this study, dependability will be achieved through the use of an audit trail. The researcher will create an audit trail by meticulously tracking the research methodology, recording the sequence of actions and procedures, and detailing influences on data collection and analysis. This will include archiving relevant documents such as research procedures, interviews, raw data, manuscripts, and field notes.

- Conformability

Korsjens and Moser (27) define conformability as “the extent to which other researchers can verify the results of the study.” In this study, the researcher will strive to ensure that the findings reflect the experiences and perspectives of the participants, rather than the researcher’s own biases or preferences.

### 3.12 Phase 1c: synthesis and integration of the study findings

The synthesis of findings involves collecting and merging information and results of a particular topic (28). The researcher will use SWOT analysis to synthesize the findings. SWOT is known as a method for assessing the Strengths, Weaknesses, Opportunities, and Threats associated with a particular project (29). The researcher will use SWOT analysis to synthesize the study’s findings by identifying key strengths, weaknesses, opportunities, and threats related to the development of a mental health awareness program. Strengths and weaknesses will focus on internal factors that support or hinder the program’s success, while opportunities and threats will examine external elements that could enhance or challenge the initiative. This structured approach will guide the development of program objectives and activities to ensure a well-informed and effective intervention. If there are any gaps to be addressed after quantitative approach, the researcher will integrate the findings of quantitative and qualitative approaches. To integrate the results of this study, the researcher will first collect and analyze quantitative data, then proceed with the collection and analysis of qualitative data. The findings from both phases will be combined and synthesized into a cohesive single study.

**Integration of the Study Results and Findings:** if there are any gaps to be addressed after quantitative approach, the researcher will integrate the findings of quantitative and qualitative approaches. To integrate the results of this study, the researcher will first collect and analyze quantitative data, then proceed with the collection and analysis of qualitative data. The findings from both phases will be combined and synthesized into a cohesive single study.

#### 3.12.1 Phase 2: development of a program

**Figure 2:**
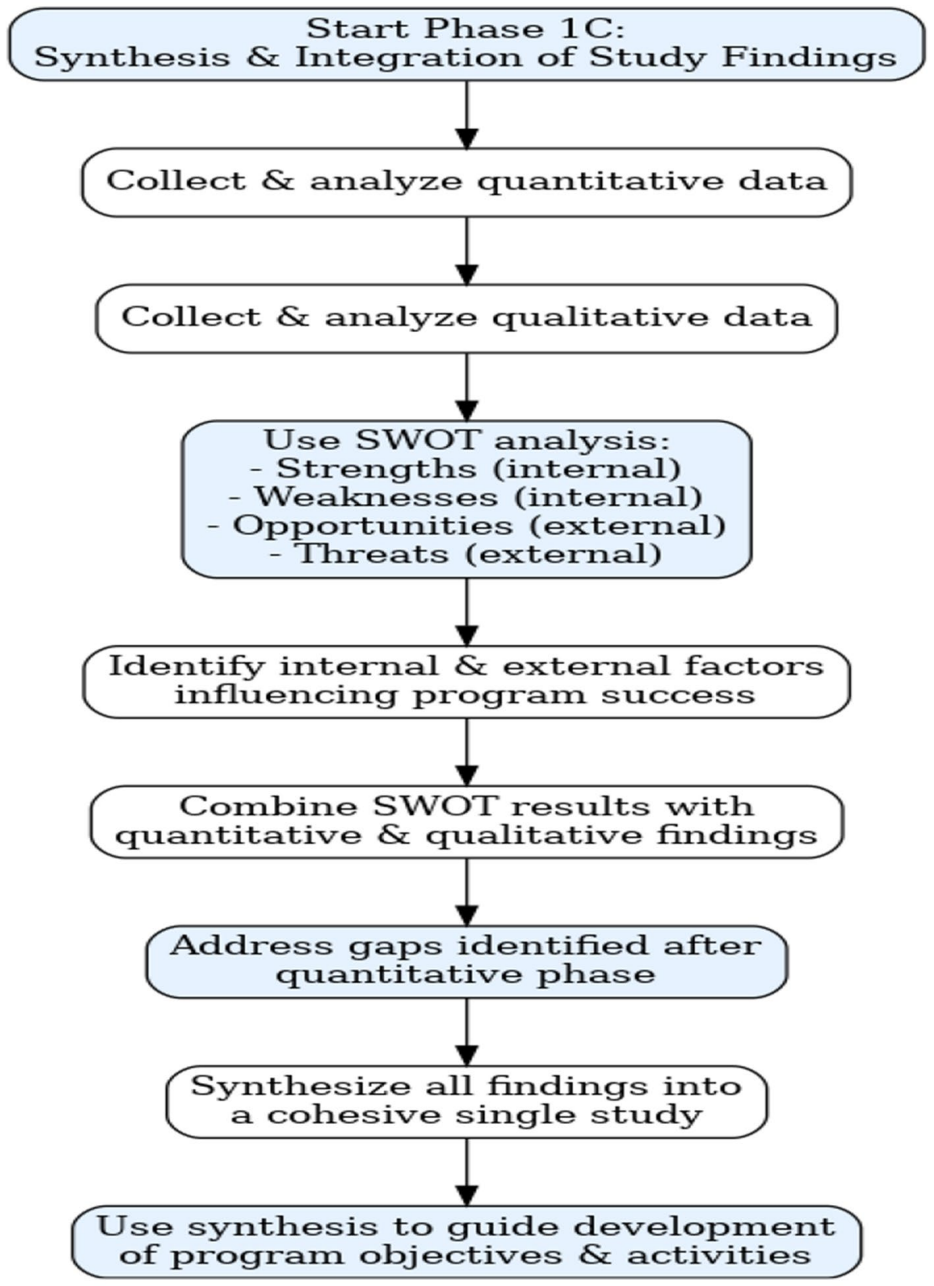
Summary synthesis and integration of the study findings.

**Figure 3:**
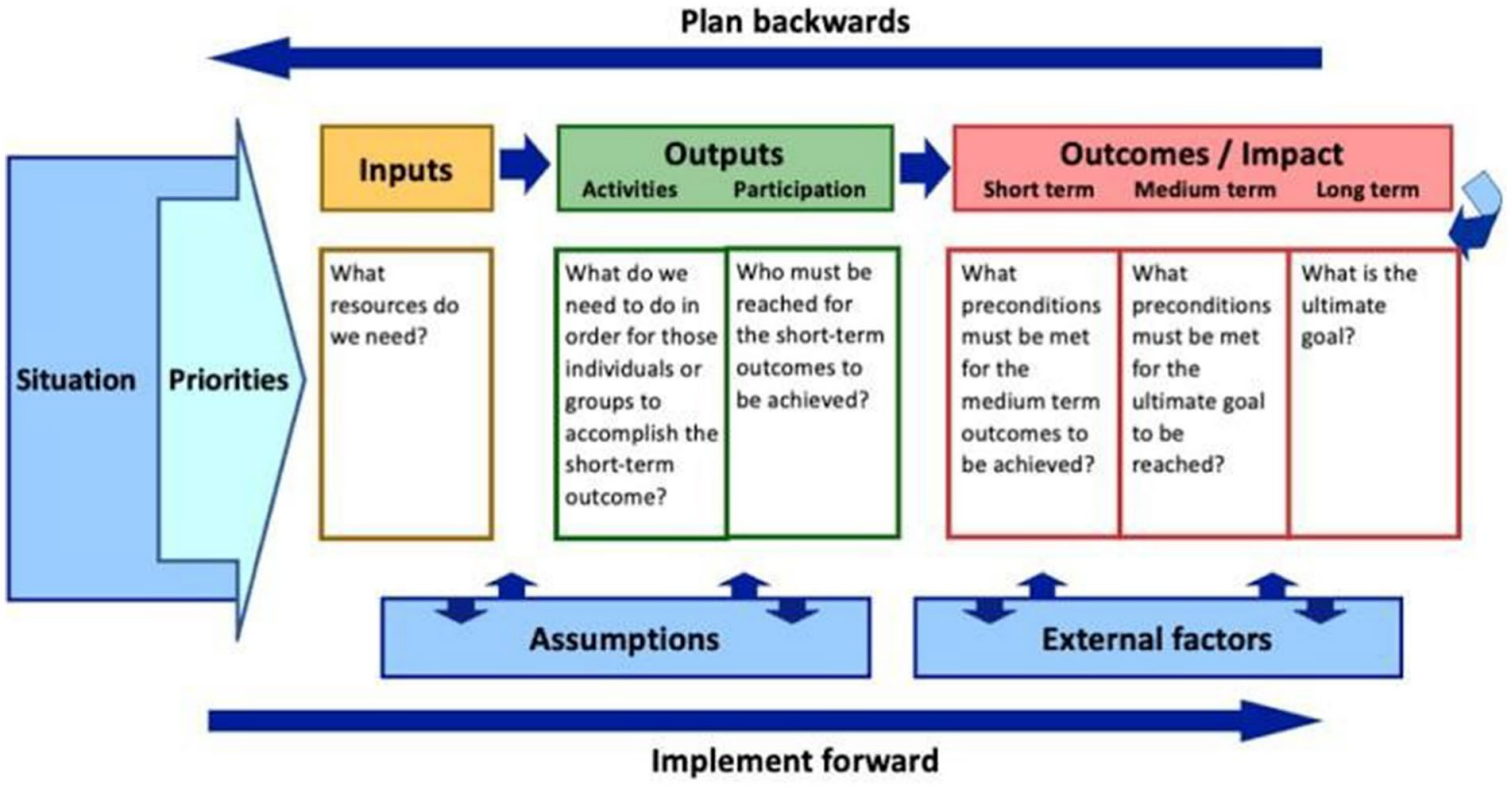
Logic model diagram.

This phase’s objective is to develop a mental health awareness program for adolescents in rural secondary schools in Mopani District, Limpopo Province, using a logic model as the guiding framework. The logic model provides a structured approach for planning, implementing, and evaluating the program by outlining key components: inputs (resources needed), activities (planned actions), outputs (immediate results such as sessions held or materials distributed), and outcomes (changes in knowledge about mental health, attitudes, or behavior; see Figure 2) (30). The program aims to enhance mental health awareness, promote early detection and intervention, and improve learner engagement and understanding of mental health issues. Objectives will be clearly formulated based on these goals to ensure meaningful and measurable results. The development of the program will follow a logic model. Furthermore, after the SWOT analysis of the study, the Build, Overcome, Explore, and Minimize (BOEM) model will be adopted. BOEM model will be adopted to build strengths, overcome weaknesses, exploit opportunities, and mitigate threats identified. The mental health awareness program will be presented in a table format (Figure 3).

Furthermore, after the SWOT analysis of the study, the Build, Overcome, Explore, and Minimize (BOEM) model will be adopted. BOEM model will be adopted to build strengths, overcome weaknesses, exploit opportunities, and mitigate threats identified. The mental health awareness program will be presented in Table 2.

**Table 2:** The proposed logic model for implementation of the program.

SWOT Situational factors	Goal	objectives	Inputs	BOEM Activities	outputs	Outcomes	Impact
							

#### 3.12.2 Phase 3: validation of the developed program

This phase’s objective is to validate the developed program. To validate the program developed the researcher will employ Delphi technique. Delphi technique refers to an interrogation process that allows for decisions to be made by consulting experts in the field of interest (31). The researcher will use the Delphi technique to validate the developed program by consulting a panel of 12 experts in mental health, education, and rural development. Through multiple rounds of structured questionnaires developed by the reseacher, experts will review the program and provide feedback until consensus is reached. Experts will be selected through purposive sampling, and their responses will be analyzed both qualitatively and quantitatively. Validity and reliability will be ensured through clear documentation, standardized procedures, and anonymous feedback. The program will be refined after each round based on expert input (Table 3).

**Table 3:** Methods summary.

Phases	Phase 1		Phase 2	Phase 3
	Research methodology		Program development	Program validation
Design	Explanatory sequential mixed methods design			
Approach	Quantitative	Qualitative		
Objectives	Objective/s 1–4		Objective 4	Objective 5
Participants	Adolescents Teachers	Adolescents Teachers		Psychologists, registered counselors and social workers
Sampling of schools	Cluster sampling		Logic model	Delphi model
Sampling of adolescents	Systematic sampling	Purposive and snowball sampling		
Sampling of teachers	Simple random sampling	Purposive and snowball sampling		
Data collection	Structured questionnaires	Semi-structured interviews		
Data analysis	SPSS	Thematic data analysis		

## 4 Discussion

Many public secondary schools in the Mopani district of Limpopo, South Africa, lack regular visits from health practitioners, leading to delayed identification and intervention for adolescents facing mental health challenges. The absence of structured mental health programs and awareness initiatives in these schools exacerbates the issue, allowing stigma and substance abuse to persist while discouraging help-seeking behavior among students. This gap in service provision contributes to rising cases of anxiety, depression, stress, and poor coping mechanisms, all of which hinder students’ academic performance and overall well-being. In response, this study aims to develop an intervention program to promote mental health awareness among adolescents in rural-based secondary schools, aligning with Sustainable Development Goals (SDGs) 3 and 4, which focus on health and inclusive education. The findings might support the creation of a tailored mental health promotion strategy and offer guidance for policymakers, educators, and mental health professionals.

## Ethics statement

Ethical considerations for this study included obtaining ethical clearance from the University of Limpopo and checking for plagiarism using Turnitin. Permission to conduct the study will was sought from the Limpopo Department of Education, the Mopani district office, and principals of the selected schools. Written informed consent was obtained from the participants parents/guardians, and assent from adolescents for participation in the study.
